# Magnitude of risk factors and in-hospital mortality of stroke in Ethiopia: a systematic review and meta-analysis

**DOI:** 10.1186/s12883-020-01870-6

**Published:** 2020-08-19

**Authors:** Muluneh Alene, Moges Agazhe Assemie, Leltework Yismaw, Daniel Bekele Ketema

**Affiliations:** Department of Public Health, Debre Markos University, Debre Markos, Ethiopia

**Keywords:** Mortality rate, In-hospital, Stroke, Meta-analysis

## Abstract

**Background:**

The morbidity and mortality of stroke is disproportionately high in developing countries owing to the poor health care system and poor neurologic interventions. Though a number of studies were conducted to estimate the in-hospital mortality rate of stroke in Ethiopia, the lack of a nationwide study that determines the overall magnitude of risk factors and in-hospital mortality rate of stroke is an important research gap. Meta-analysis is key to improve the accuracy of estimates through the use of more data sets. Thus, this study was aimed to determine the overall magnitude of risk factors and in-hospital mortality rate of stroke in Ethiopia.

**Methods:**

This study was conducted following the PRISMA checklist. We searched from Google Scholar, PubMed, Science Direct, Web of Science, CINAHL, and Cochrane Library databases for studies. Each of the original studies was assessed using a tool for the risk of bias adapted for cross-sectional studies. Data were pooled and a random effect meta-analysis model was fitted to provide the overall magnitude of risk factors and in-hospital mortality rate of stroke. Also, the subgroup analyses were performed to examine how the in-hospital mortality rate varies across different groups of studies.

**Results:**

In this study, the overall magnitude of hypertension, diabetes mellitus, and atrial fibrillation among stroke patients were 47% (95%CI: 40–54), 8% (95CI%:6–12), and 10% (95%CI: 5–19), respectively. The overall in-hospital mortality of stroke in Ethiopia was 18% (95%:14–22). The highest magnitude of in-hospital mortality of stroke was observed in SNNPR and the lowest was noted in Tigray region. In addition, the magnitude of the in-hospital mortality rate of stroke was 15.1% (95%CI: 11.3–19.4), and 19.6%(95%CI: 14.1–25.7), among studies published before and after 2016, respectively.

**Conclusions:**

Our pooled result showed that nearly one-fifth of stroke patients have died during hospitalization. The most common risk factor of stroke among the included studies was hypertension followed by atrial fibrillation and diabetes mellitus. There is a need for a better understanding of the factors associated with high blood pressure, especially in countries with a high risk of stroke.

## Background

The incidence, prevalence, and mortality rate of stroke have been increased worldwide, with most of the burden being in low and middle-income countries [1, 2]. Globally, it is ranked as the second leading cause of death with annual mortality rate of 5.5 million, and it is now the leading cause of physical disability in peoples aged 65 years and above [3, 4]. The burden of stroke lies not only in high mortality but also high morbidity resulting in up to half of the survivors being chronically disabled [3, 5, 6]. Nearly one-fifth (17%) of people worldwide will have a stroke in their lifetime. Developing countries have a higher burden of non-communicable diseases than the rest of the world [7]. More-than two-third (70%) of strokes occur in low- and middle-income countries [8].

Despite the fact that Ethiopia is progressing towards universal health coverage, the country faces the double burden of both communicable and non-communicable diseases [9]. According to WHO data published in 2017, stroke deaths in Ethiopia reached 6.23% of total deaths. In addition, the age-adjusted death rate of stroke in the country is 89.82 per 100,000 of the population. Previous reports that showed the future trend of stroke in SSA revealed that stroke burden will increase over the coming years owing to poor healthcare seeking behavior, and poor neurologic interventions [10]. In addition, existing studies that projected the burden of stroke in SSA between 2008 and 2025 showed that the number of people with hypertension will escalate by more-than two-third [11]. Even though, stroke is a common health problem affecting the lives of many people worldwide, its burden and risk factors are different according to geographical variation [12]. Besides, previous reports indicated that 90% of the burdens of stroke are attributable to modifiable risk factors [12]. Of this, three-quarters of stroke burden is attributable to behavioral risk factors [13].

Though a number of studies were conducted to estimate the magnitude of risk factors of stroke and in-hospital mortality rate in Ethiopia, the unavailability of a nationwide study that determines the magnitude of risk factors and in-hospital mortality rate of stroke is an important research gap. Meta-analysis is key to improve the accuracy of estimates through the use of more data sets. Thus, this study was aimed to determine the overall magnitude of risk factors of stroke and in-hospital mortality rate in Ethiopia.

## Methods

### Study setting

Ethiopia is situated in the horn of Africa, and bordered by Eritrea to the north, Sudan and South Sudan to the west, Kenya to the south, and Djibouti and Somalia to the east. The total population in the country is estimated to be more than one hundred ten million, and of which more than 84% live in rural areas. Ethiopia is a Federal Democratic Republic composed of 9 National Regional states: namely Tigray, Afar, Amhara, Oromia, Somali, Benishangul-Gumuz, Southern Nations Nationalities and People Region (SNNPR), Gambella and Harari, and two Administrative states (Addis Ababa City administration and Dire Dawa city council) [14]. Stroke care at the acute phase of the disease in Ethiopia is being done in hospitals preferably in an intensive care units [15].

### Eligibility criteria

The eligibility of the study was determined using the following criteria:

- Study design: all facility-based observational studies
- Study setting: all studies conducted in Ethiopia.
- Outcome: all studies reporting either the magnitude of risk factors of stroke or in-hospital mortality of stroke
- Articles: Both published and unpublished studies

### Searching for studies

The comprehensive search for studies was done by two (MA and LY) of the authors between October, 15, and November 30, 2020. Google Scholar, PubMed, Web of Science, Science Direct, and CINAHL databases were searched for studies. First, articles were searched by examining the full titles (“Magnitude of risk factors and in-hospital mortality of stroke in Ethiopia”) and then keywords (stroke in-hospital outcome, risk factors of stroke, Ethiopia). These keywords were used separately and in combination using Boolean operators “OR” or “AND”. In addition, we searched from the reference lists of all the included studies (snowball technique) to identify any other studies those may have been missed by our search strategy. Finally, all studies were imported into reference management software (Mendeley desktop).

### Data extraction

All essential data from the original studies were extracted independently by two (MA and LY) of the authors. The following information was identified for the purpose of data extraction: The last name of the first author and year of publication, the region of the study conducted, study design and period, sample size, stroke type, the magnitude of risk factors, and in-hospital mortality rate. In this study, we considered both types of ischemic (thrombotic and embolic) and hemorrhage (Intracerebral and Subarachnoid) stroke. Any inconsistencies in the data extraction process were decided through discussion involving all authors.

### Quality assessment tool

Two reviewers (MA and MAA) assessed the quality of the included articles. The Newcastle-Ottawa Scale (NOS) adapted for cross-sectional studies was used to evaluate the quality of studies [16]. This tool was organized from three major sections. Consequently, the first section scored on the basis of one to five stars focuses on the methodological quality of each study. The second segment of the tool evaluates the comparability of the study groups with a maximum possibility of two stars to be given.

The last section of the tool is concerned with the outcomes and statistical analysis of the included studies with a maximum possibility of three stars to be given. Any inconsistent report between the two reviewers (MA and MAA) was decided by taking the average score of the two reviewers’. Finally, the assessed articles with a score of less than six out of ten were considered as achieving low quality.

### Data analysis

After extracting all relevant data using Microsoft excel software, data were exported to R 3.6.1 version software for meta-analysis. The double arcsine transformation which stabilizes the sampling variance was applied to estimate the weighted average magnitude of risk factors of stroke and in-hospital mortality, and the transformed summary are converted back for ease of interpretation [15]. We assessed the consistency of studies using *I*^*2*^ test statistics [16]. This test evaluates the null hypothesis of all the original studies that examines the same effect. In this study, there was considerable heterogeneity between the original studies (*I*^*2*^ = 84%, *p* < 0.01). A random effect model is needed, and to account for between-study variance a random effect meta-analysis with an estimation of DerSimonian and Laird method was performed.

Univariate meta-regression was employed to explore how the study characteristics associated with the outcome of interest. In addition, the subgroup analyses were performed to investigate how the in-hospital mortality of stroke varies across different groups of studies. Furthermore, a sensitivity analysis was done to identify influential articles.

## Results

### Description of studies

**Fig. 1:**
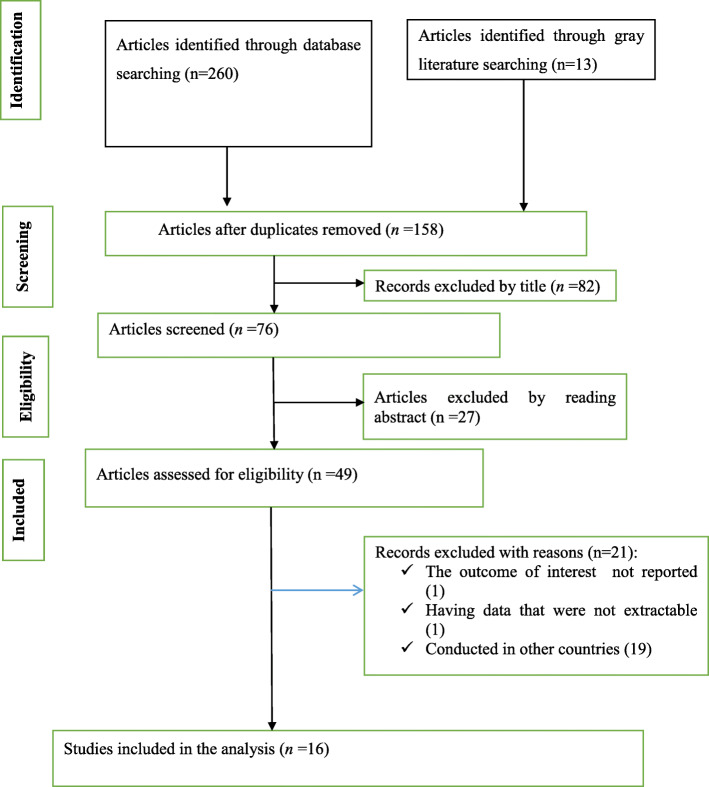
Flow chart diagram that describes the selection of studies included in the systematic review and meta-analysis of magnitude of risk factors and in-hospital mortality rate of stroke in Ethiopia

**Fig. 2:**
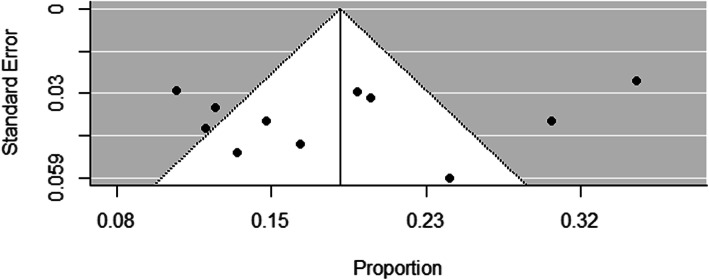
Funnel plot of the in-hospital mortality rate of stroke in Ethiopia

The flow chart diagram that describes the selection of studies included in this study is presented in (Fig. 1). In our search, a total of 260 published and 13 Gy literatures were identified. After articles were removed by duplications, title, and reading the abstract, 49 studies were assessed for eligibility criteria. Consequently, ten studies were excluded due to the outcome of interest was not reported, having data that were not extractable and conducted in other countries. Finally, a total of 16 studies that satisfy the eligibility criteria were included in this systematic review and meta-analysis. The detailed descriptions of the included studies are presented in (Table 1). The publication year of the included studies was between 2015 and 2019. Of the total included studies, three studies were prospective facility-based studies, while the other nine studies were retrospective facility-based studies. Our summary risk of bias assessment showed that more-than two-third (68.8%) of the studies had a low risk of bias, while 31.2% of studies had a high risk of bias. The issue of publication bias was evaluated by visual inspection of the funnel plot and using Egger’s regression test. Though the funnel plot looks asymmetrical (Fig. 2), the Egger’s test showed that no relationship between the effect size and its precision (*P*-value = 0.63).

**Table 1:** Characteristics of the included studies conducted in Ethiopia on magnitude of risk factors and in-hospital outcome of stroke

First author and publication year	Region	Study design	Study period	Sample size	In-hospital mortality rate	Types of stroke	
						Ischemic	Hemorrhagic
Asefa et al. (2018) [17]	Oromiya	RCS	December 2015 to November 2017	394	37.87%	35.69%	64.31%
Betero et al. (2019) [18]	Oromiya	IBCS	March 2016 to May 2019	111	16.2%	80.1%	18.2%
Deresse et al. (2015) [19]	SNNPR	prospective study	May 2013 to April 2014	163	14.7%	49.7%	50.3%
Erkabu et al. (2018) [20]	Amhara	Retrospective hospital-based	February 2014 to August 2016	303	11%	59.4%	40.6%
Fekadu et al. (2019) [21]	Oromiya	Retrospective	from 2013 to 2017	364	NR	52.7%	NR
Fekadu et al. (2019) [21]	Oromiya	Prospective	March 10 to July 10, 2017	116	NR	51.7%	48.3%
Gebremariam et al. (2016) [22]	Tigray	Retrospective case study	March 2012 to February 2014	142	12.0%	55.6%	32.4%
Gebreyohannes et al. (2019) [23]	Amhara	Retrospective cohort	November 2012 and September 2016.	229 Ischemic stroke	12.5%	NR	NR
Gedefa et al. (2017) [24]	Addis Abeba	hospital based retrospective study	September 2015 to August 2016	163	30.1%	35.6%	61.3%
Greffie et al. (2015) [25]	Amhara	Retrospective chart record analysis	June 2010 to May 2013	98	13%	69.4%	30.6%
Mamushet et al. (2015) [26]	Addis Abeba	Prospective	June 2008 to March 2009	71	23%	NR	NR
Kassaw et al. (2018) [27]	Addis Abeba	Hospital based RCS	July 2015 to February 2018	256	20%	51.2%	37.6%
Kefale et al. (2019) [18]	Oromiya	CS	March 30/2016 to May30/2019	111	16.2%	80.1%	18.2%
Sultan et al. (2017) [28]	Addis Ababa	RCS	December 2010 to December 2014	301	19.2%	54%	46%
Temesgen et al. (2018) [29]	Oromiya	RCS	March 2012 to March 2017	73	NR	65.8%	21.9%
Zewdie et al. (2018) [30]	Addis Ababa	CS	August 2015 to January 2016	104	NR	44%	56%

*CBCS* Community Based Cross Sectional Study, *EDHS* Ethiopia Demographic and Health Survey, *HDSS* Health and Demographic Surveillance System, *IBCS* Institution Based Cross Sectional Study, *KDS-HRC* Kersa Demographic Surveillance and Health Research Center, *NR* Not Report, *SNNRP* Southern Nations, Nationalities, and Peoples’ Region

### Magnitude of risk factors of stroke in Ethiopia

**Fig. 3:**
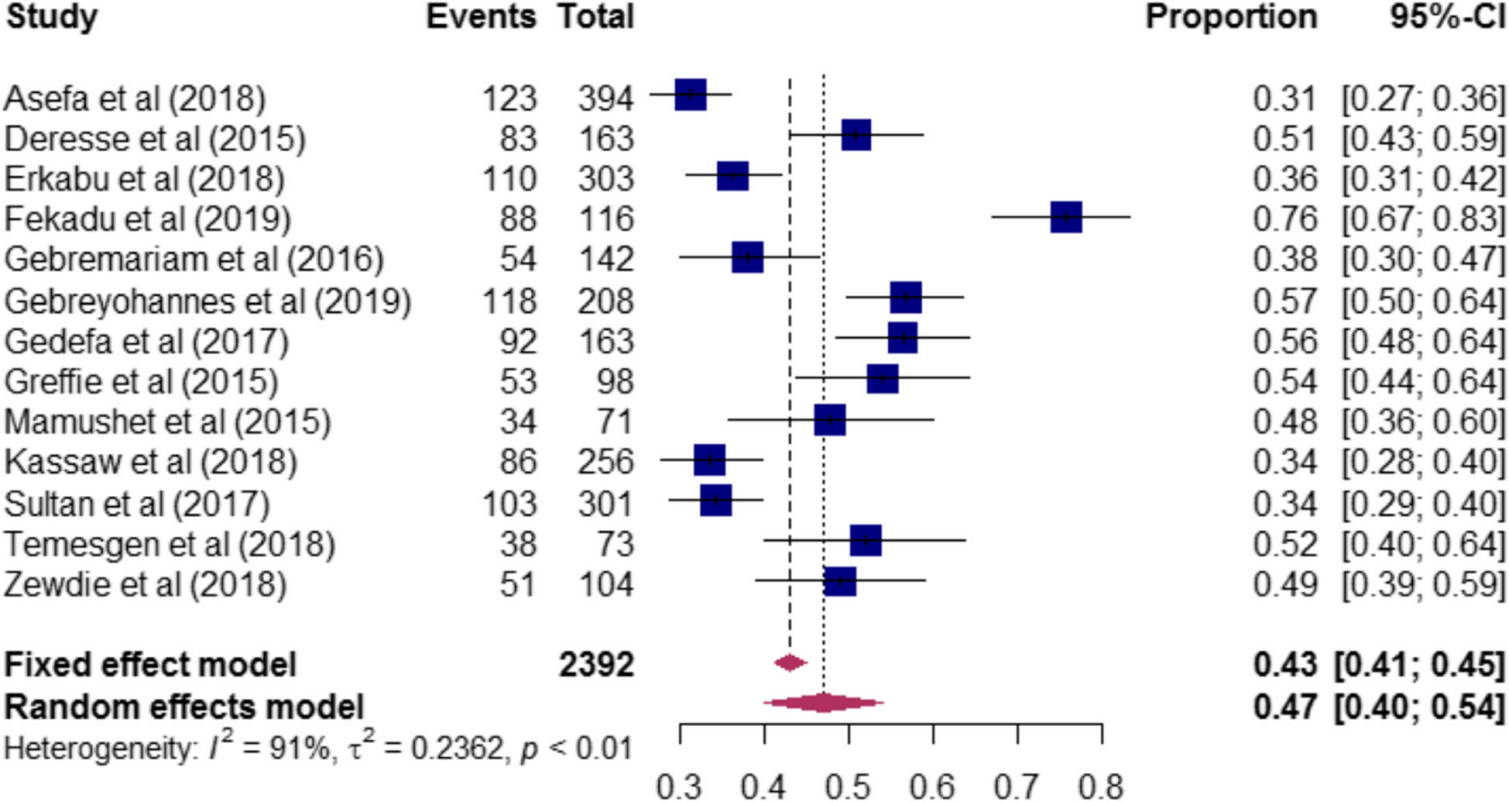
The proportion of hypertension among stroke patients included in the systematic review and meta-analysis

**Fig. 4:**
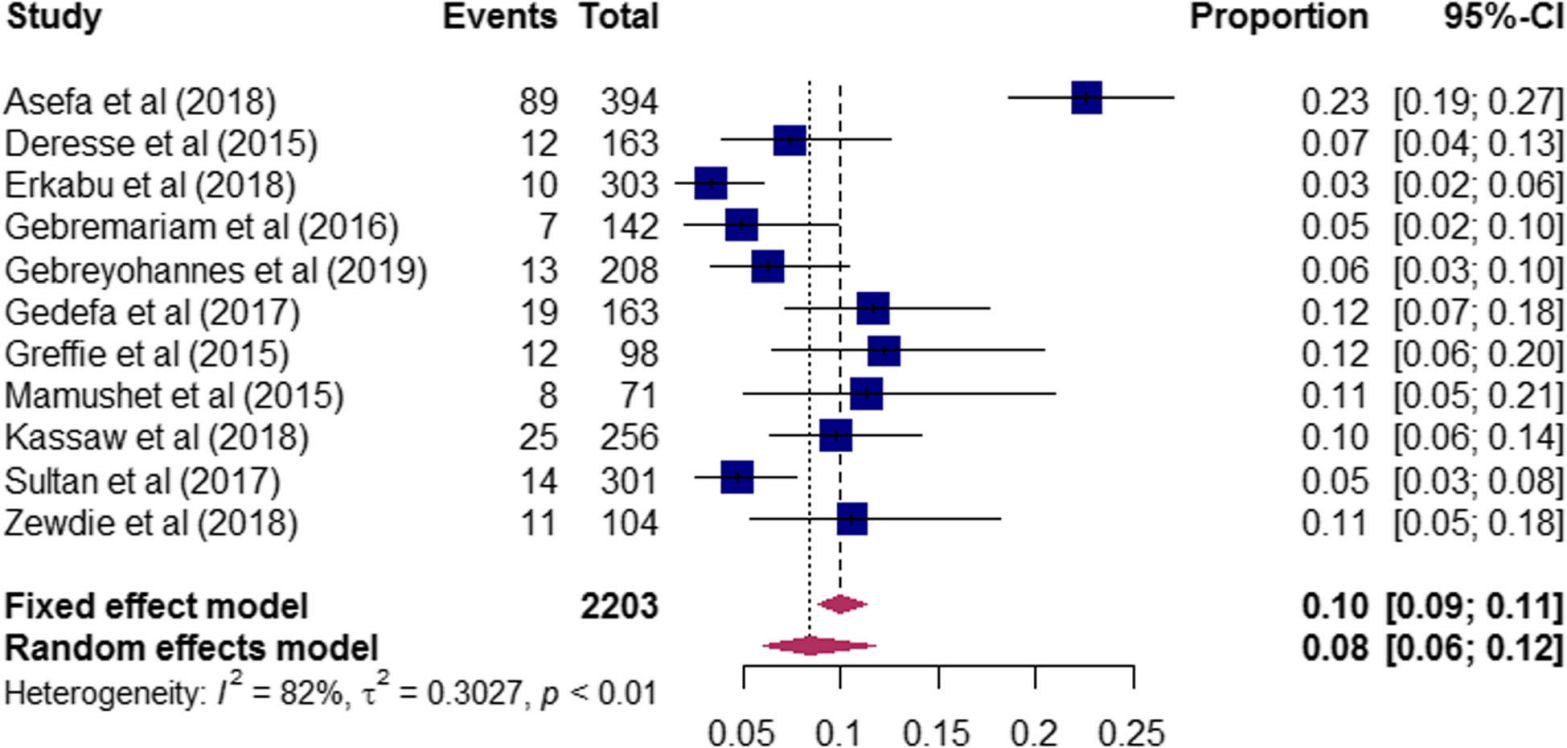
The proportion of Diabetes Mellitus among stroke patients included in the systematic review and meta-analysis

**Fig. 5:**
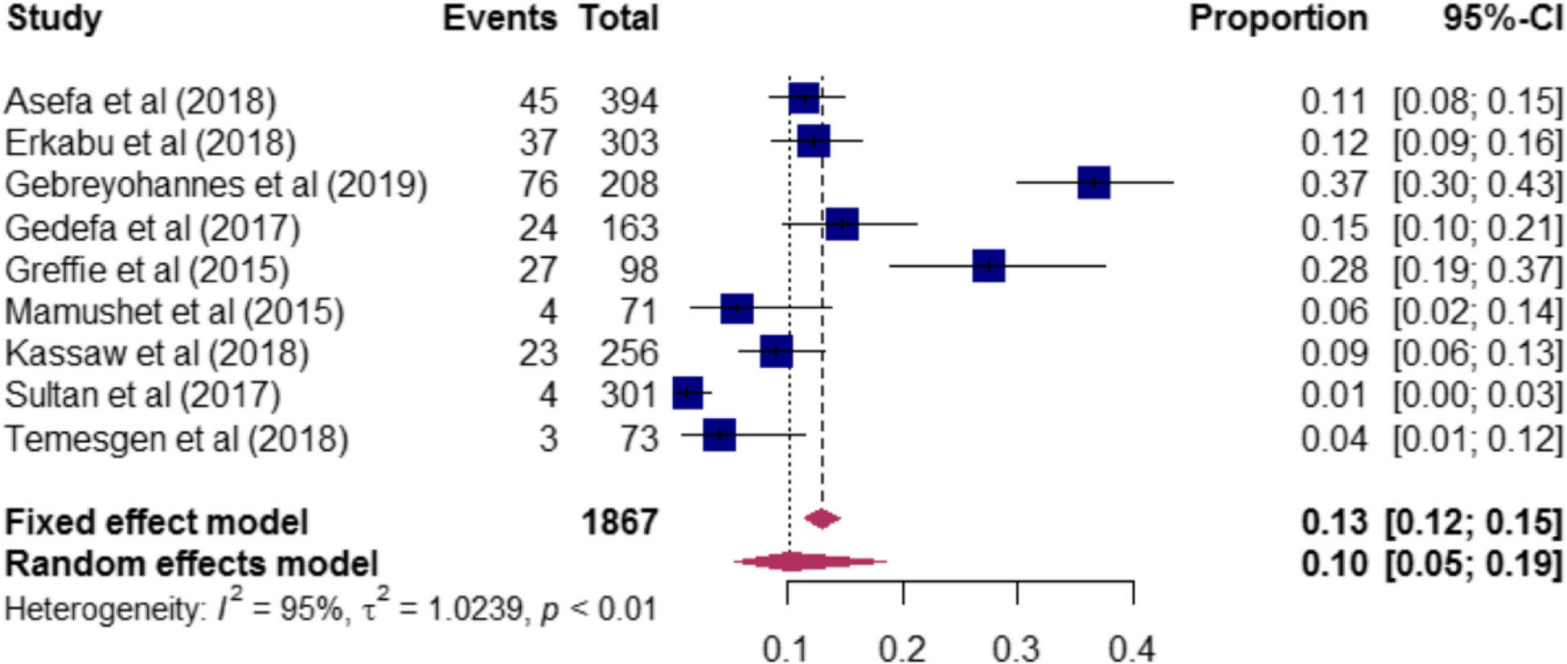
The proportion of atrial fibrillation among stroke patients included in the systematic review and meta-analysis

The magnitude of risk factors of stroke among the included studies are presented in (Table-S1). The proportion of hypertension among stroke patients ranged from 31% [17] to 76% [21]. A total of 2, 392 stroke patients were included to determine the pooled magnitude of hypertension among stroke patients. Accordingly, our pooled analysis showed that nearly half [47% (95%CI: 40–54)] of stroke patients had hypertension (Fig. 3). In this review, stroke patients who had diabetes mellitus ranged from 3% [20] to 23% [17]. To determine the pooled magnitude of diabetes mellitus among stroke patients, a total of 2203 patients were included. Consequently, we found that the overall magnitude of diabetes mellitus among stroke patients was 8% (95CI%:6–12) (Fig. 4). Furthermore, the proportion of atrial fibrillation among stroke patients included in this study ranged from 1% [28] to 37% [23], and our meta-analysis revealed that one-tenth [10% (95%CI: 5–19)] of stroke patients had atrial fibrillation (Fig. 5).

### In-hospital mortality of stroke in Ethiopia

**Fig. 6:**
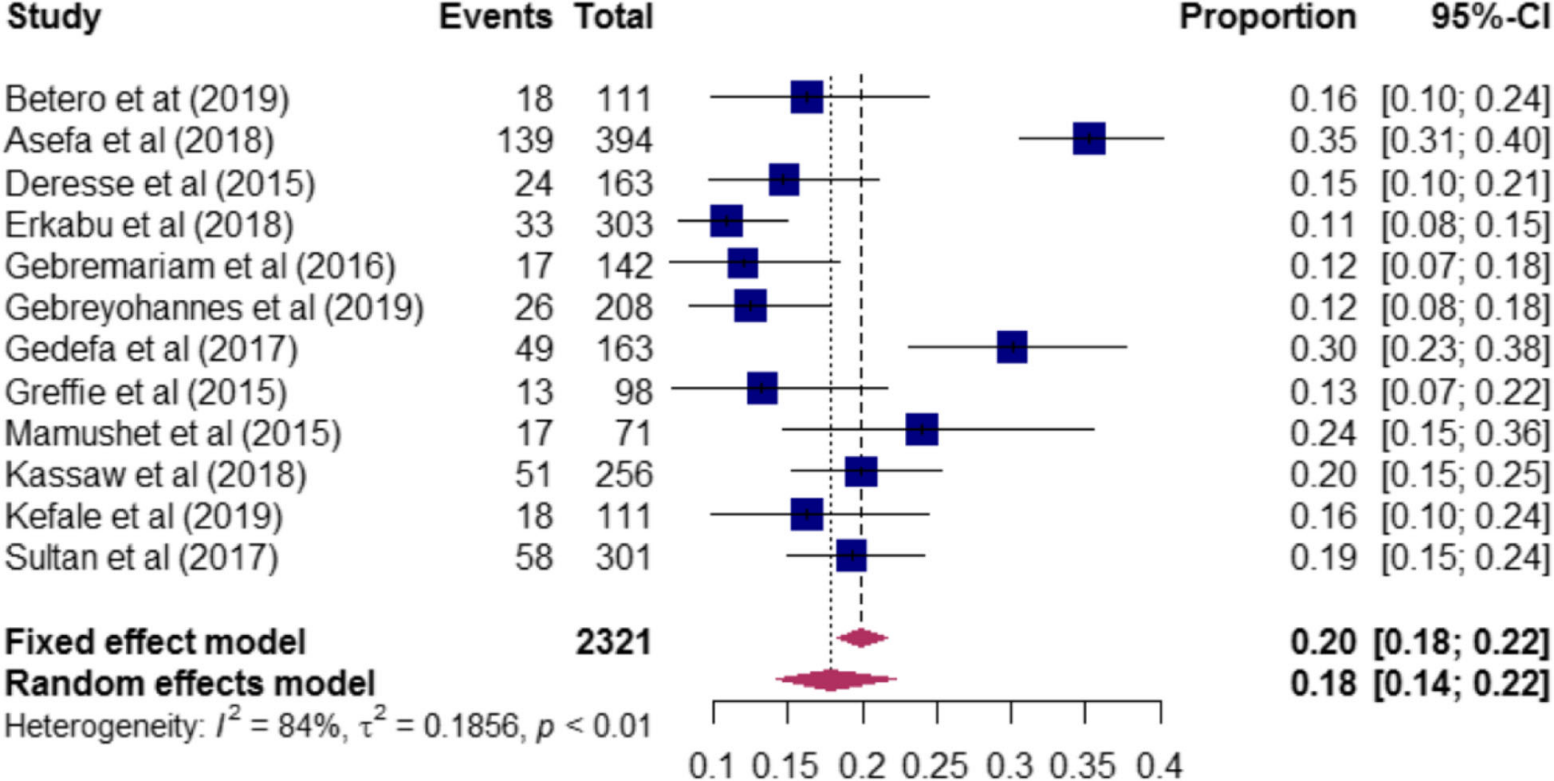
In-hospital mortality rate of stroke among included studies in Ethiopia

In-hospital mortality of stroke among the included studies varies from 11% [20] to 38% [17]. A total of 2321 stroke patients were included to determine the pooled magnitude of in-hospital mortality among stroke patients, and the pooled in-hospital mortality of stroke in Ethiopia was 18% (95%:14–22) (Fig. 6).

The result from univariate meta-regression showed that in-hospital mortality of stroke varies by regions of the study conducted and quality score (Table 2). The subgroup analyses that shows how the magnitude of in-hospital mortality of stroke differs across different subgroups of studies are presented in (Table 3). Accordingly, the highest magnitude of in-hospital mortality of stroke was observed in Southern Nation’s Nationalities and Peoples Region (SNNPR), and the lowest was noted in Tigray region. The subgroup analysis by publication year showed that the magnitude of the in-hospital mortality rate was 19.6%(95%CI: 14.1–25.7) for studies published after 2016. Similarly, the magnitude of in-hospital mortality rate of stroke was 15.1% (95%CI: 11.3–19.4) for studies published in 2016 and before.

**Table 2:** Subgroup analysis of studies included in meta-analysis on in-hospital outcome of stroke in Ethiopia

Subgroup	Included studies	In-hospital mortality rate
**Sample size**		
≤ 163 (less-than median)	7	17.7% (95% CI:13.2–22.9)
> 163 (greater-than median)	5	18.9% (95%CI:11.5–27.7)
**Region**		
Amhara	3	14.7% (95%CI:9.6–20.6)
Oromiya	3	10.8% (95%CI:7.8–14.6)
SNNPR	1	35.2% (95%CI:30.6–40.0)
Addis Ababa	3	16.3% (95%CI:9.8–23.8)
Tigray	1	10.7% (95%CI:7.5–14.5)
**Publication year**		
2016 and before	4	15.1% (95%CI:11.3–19.4)
After 2016	8	19.6% (95%CI:14.1–25.7)
**Quality score**		
low	5	16.3% (95%CI:10.0–23.8)
High	7	35.2% (95%CI:30.5–40.0)

**Table 3:** Related factors with heterogeneity of in-hospital mortality of stroke (based on univariate meta-regression)

Sources of heterogeneity	Coefficient	*P*-value
Sample size	0.0003	0.26
Publication year	0.0014	0.94
Quality score		
Low	0.06	0.25
High	0.42(constant)	< 0.0001
Religion		
Amhara	−0.1604	0.0175
Oromiya	−0.0287	0.6495
SNNPR	−0.1222	0.1991
Tigray	−0.1617	0.0933
Addis Ababa	0.5190 (constant)	< 0.0001

To assess the robustness of results, first, articles which have larger than two in the absolute value of studentized residuals were screened. In the screening process article [17] seems influential (Figure S1). Next, leave-one-out sensitivity analysis has been performed to determine whether or not they are truly influential, and it indicates that the effect is not statistically significant (Figure S2).

## Discussion

This study was aimed to determine the overall magnitude of risk factors and in-hospital mortality of stroke in Ethiopia. We observed that hypertension is the most common risk factor of stroke among the included studies. Our meta-analysis showed that nearly half (47%) of stroke patients had hypertension. Previous evidence also showed that people with hypertension are nearly four times more likely to have a stroke [31]. In addition, it has been shown that lowering blood pressure can decrease the risk of stroke by 30 to 40% [32]. Though hypertension is the main reported risk factor of stroke among the included studies, the proportion of hypertension among stroke patients found in the current study is lower than previous studies conducted in India, Bosnia-Herzegovina, Zambia, Nigeria, Malawi, and Bangladesh [33–38]. The possible explanation for this variation might be due to the lack of diagnostic modalities and proficiency [39]. Additionally, this could be due to the included studies measured hypertension in the medical record prior to the occurrence of stroke [22].

Diabetes is a well-established risk factor of stroke, and our analysis showed that diabetes mellitus is the second most common comorbidity of stroke. Nearly one-tenth (8%) of stroke patients had diabetes mellitus. This is due to the fact that diabetes causes various microvascular and macrovascular changes often ending in major clinical complications [40]. This result is comparable with a previous study conducted in SSA [41]. However, this proportion of diabetes mellitus among stroke patients is lower than a study conducted in Nigeria and Egypt [42, 43].

The in-hospital mortality rate of stroke was the main outcome of interest, and the result found in this study showed that the overall in-hospital mortality rate of stroke in Ethiopia was 18% (95%:14–22). This result is comparable with other hospital-based studies conducted in sub-Saharan Africa, Cameroon, Nigeria, and Kenya [41, 44–46]. However, this result is lower-than previous studies conducted in Ghana, Tanzania, Gambia, Uganda, and Burkina Faso [47–51]. The in-hospital mortality rate of stroke was 41.1, 33.3, 41, 66.7, and 28.7% in Ghana, Tanzania, Gambia, Uganda, and Burkina Faso, respectively. The possible reason for this discrepancy could be explained by the low proportion of hypertension among stroke patients. Hypertension is usually associated with a hemorrhagic stroke which has a higher mortality rate [52]. Ischemic stroke however has a better prognosis and hypertension is not as such a very common risk factor for ischemic stroke unlike hemorrhagic stroke [53]. Hence the fact that hypertension being a less risk factor for stroke in Ethiopia, and the lower mortality can be possibly explained with this correlation. The other possible reason for this variation might be the difference in in-patient stroke care [54].

The in-hospital mortality rate of stroke in the current study was higher than previous studies conducted from Taiwan, China, Spain, and Germany [55–58]. In these countries, the in-hospital mortality of stroke was 3.1, 2.3, 7.13, and 4.9%, respectively. This variation could be explained by the advancements in stroke care and prevention, in developed countries [59].

The subgroup analyses by publication year of studies showed that the overall in-hospital mortality rate of stroke was higher among studies published after 2016. This result is supported by a study conducted in Ghana, which reports that the rates of stroke mortality for the past three decades have increased steadily [47]. The possible reason for this trend might be the demographic transitions of populations in developing countries [9, 60]. The other possible reason might be due to the rise of non-communicable diseases in developing countries, including Ethiopia. Urbanization and changes in lifestyle in the developing world will remain to rise the burden of stokes in the upcoming years [61]. The highest magnitude of in-hospital mortality was observed in SNNPR, while the smallest magnitude of in-hospital mortality was observed in Tigray region. This difference might be explained by the limited access to hospital care, paucity of staff, and shortage of facilities for diagnosis.

### Limitations

This study was not free from limitations. There is considerable heterogeneity across the included studies. The observed heterogeneity may be described by differences in the study design, the quality of the studies, and sensitivity. Our study is mainly based on the in-patient cases, and it cannot be externally validated to the general population.

## Conclusions

Our pooled result showed that nearly one-fifth of stroke patients have died during hospitalization in Ethiopia. The most common risk factor of stroke among the included studies was hypertension followed by atrial fibrillation and diabetes mellitus. In addition, this study reveals that there is an increasing trend of stroke, and its in-hospital mortality in Ethiopia. The result of this study implies that there is a need for better awareness of the risk factors associated with high blood pressure, especially in regions with a high burden of stroke, including Ethiopia. Efforts should be focused on the primary prevention of non-communicable disease and stroke.

## Supplementary information

**Additional file 1:** **Figure S1.** In-hospital mortality rate of stroke leaving out each study.

**Additional file 2:** **Figure S2.** plot of diagnostics.

**Additional file 3:** **Table S1.** Magnitude of risk factors of stroke among the included studies in Ethiopia.

**Additional file 4:** **Table S2.** Assessing the risk of bias for the included studies.

## Data Availability

All data are available in the manuscript.

## Abbreviations

PRISMA: Preferred Reporting Items for Systematic Reviews and Meta-Analyses
SSA: Sub-Saharan Africa
NOS: Newcastle-Ottawa Scale
WHO: World Health Organization
SNNPR: South Nations, Nationalities and People’s, Region
