# Supplementary figures and images for: Magnitude of risk factors and in-hospital mortality of stroke in Ethiopia: a systematic review and meta-analysis
Source: BMC Neurol. 2020 Aug 19;20:309. doi: 10.1186/s12883-020-01870-6 (PMC7437163; doi:10.1186/s12883-020-01870-6)

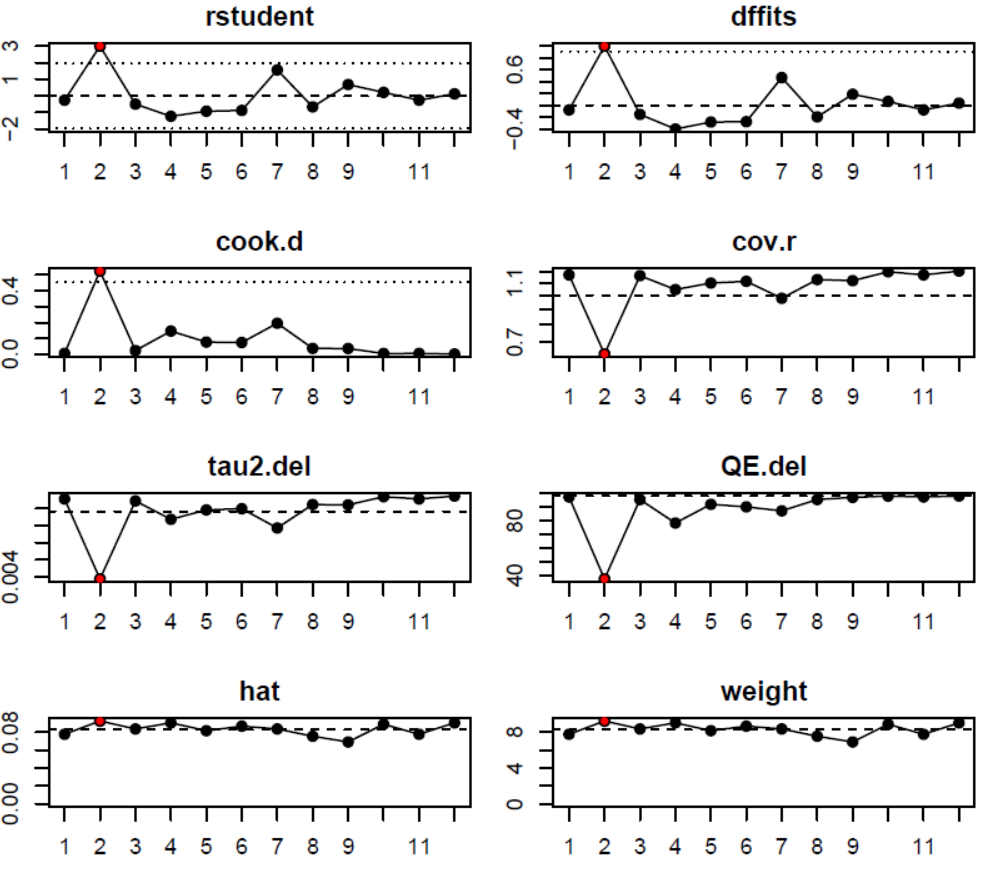

Supplement: Supplementary file 1 — Additional file 1: Figure S1. In-hospital mortality rate of stroke leaving out each study. [file 12883_2020_1870_MOESM1_ESM.docx]

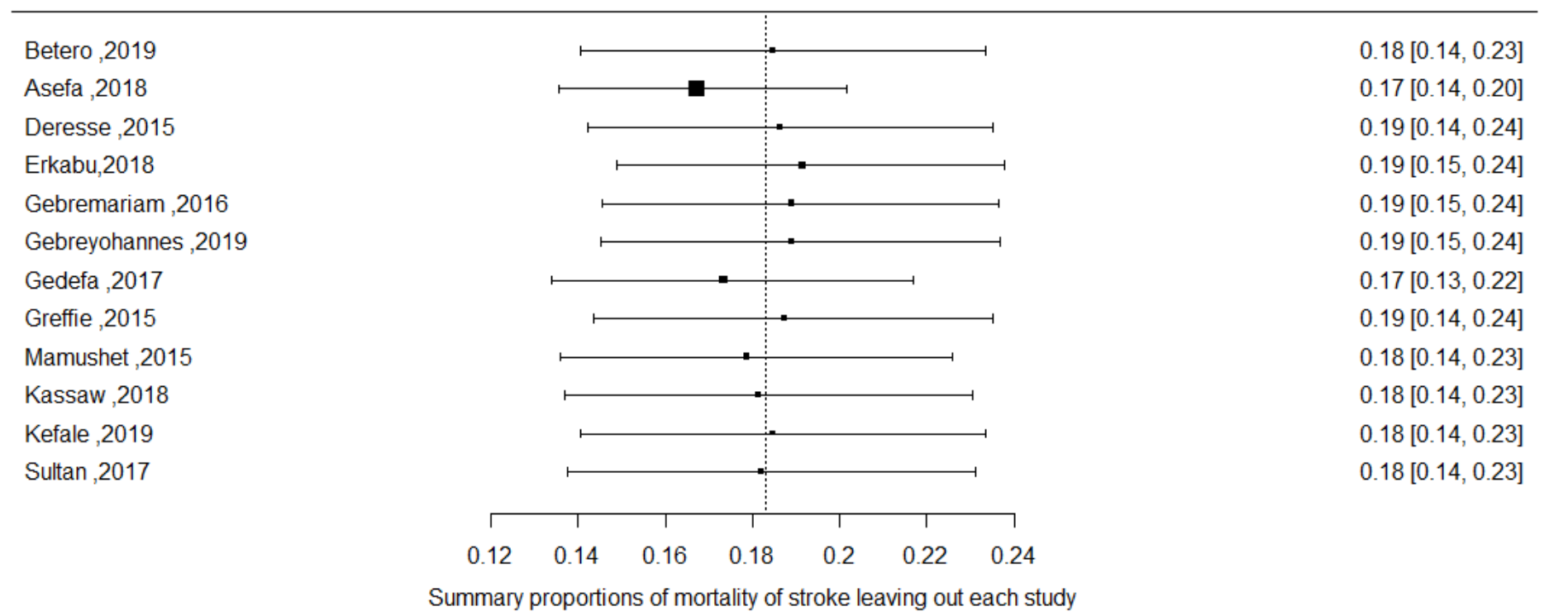

Supplement: Supplementary file 2 — Additional file 2: Figure S2. plot of diagnostics. [file 12883_2020_1870_MOESM2_ESM.docx]
