# Supplementary material for: Magnitude of risk factors and in-hospital mortality of stroke in Ethiopia: a systematic review and meta-analysis
Source: BMC Neurol. 2020 Aug 19;20:309. doi: 10.1186/s12883-020-01870-6 (PMC7437163; doi:10.1186/s12883-020-01870-6)
Supplement: Supplementary file 3 — Additional file 3: Table S1. Magnitude of risk factors of stroke among the included studies in Ethiopia. [file 12883_2020_1870_MOESM3_ESM.docx]

**Table S1**: Magnitude of risk factors of stroke among the included studies in Ethiopia.

| **First author and publication year** | **Magnitude of risk factors** |
| --- | --- |
| Asefa et al (2018) | Hypertension: 123(29.64%), Diabetes Mellitus: 89(21.45%)  Previous stroke: 66(15.91%), Atrial fibrillation: 45(10.84%)  Transient ischemic attack: 43(10.36%), Structural heart disease: 43(10.36%)  Others (HIV, RF, CHF): 6(1.44%) |
| Deresse et al (2015) | Hypertension: 83(50.9%), Cardiac diseases: 27(16.6%)  Diabetes Mellitus: 12(7.4%), Atherosclerosis: 13(38.2%)  Cigarette smoking: 8(4.9%), Current alcohol: 17(10.4%)  Previous history of alcohol intake: 12(7.4%), Previous stroke: 4(2.5%)  Transient ischemic attack: 4(2.5%), Dyslipidemia: 30(38.5%)  Family history: 2 (1.2%), TB meningitis: 5(3.1%), HIV: 2(1.9%)  Obesity: 3(1.8%) |
| Erkabu et al (2018) | Hypertension: 110(36.3%), Diabetes: 10(3.3%), Dyslipidemia: 62(20.4%)  Atrial fibrillation: 37(32.2%), Structural heart disease: 28(9.2%)  HIV: 3, Past cardiovascular accident/TIA: 11(3.6%), History of cancer: 2(0.7%), Smoking history: 0, Alcoholic: 2(0.7%) |
| Fekadu et al (2019) | Hypertension (75.9%), Family history (33.6%)  Alcohol intake (22.4%), Smoking (17.2%), Heart failure (17.2%). |
| Gebremariam et al (2016) | hypertension: 38.0%, Diabetes Mellitus: 4.9% |
| Gebreyohannes et al (2019) | Hypertension: 118 (56.7%), Diabetes Mellitus: 13 (6.3%)  Vascular disease: 61 (29.3%), Previous MI: 5 (2.4%)  AF: 76 (36.5%), CAD/IHD: 14 (6.7%)  CHF: 70 (33.7), Any VHD: 72 (34.6%) |
| Gedefa et al (2017) | Hypertension: 92 (56.4%), Diabetes Mellitus: 19 (11.6%)  Smoking: 13 (8.0%), Structural heart disease: 30 (18.4%)  Atrial fibrillation: 24 (14.7%), Transient ischemic attack: 9 (5.5%)  Previous stroke: 15 (9.2%), HIV: 1 (0.6%)  Hypertension and diabetes mellitus: 14 (8.5%) |
| Greffie et al (2015) | Hypertension:53, Diabetes mellitus:12, Structural heart disease:46  Atrial fibrillation:27, Previous stroke:7 |
| Mamushet et al (2015) | Hypertension: 34 (53.1%), Diabetes mellitus: 8 (12.5%)  Atrial fibrillation: 4 (6.3%), RVHD: 4 (6.3%)  Other CV diseases: 7 (10.9%), Smoking History: 6 (9.4%)  Renal illness: 4 (6.3%), Seizure: 2 (3.1%)  Past History of stroke: 5 (7.8%) |
| Kassaw et al (2018) | Hypertension:86, Diabetes type II:25  Other cardio-vascular diseases:28, HIV:9  Atrial fibrillation:23, Other comorbidities:27 |
| Sultan et al (2017) | Hypertension:103, Diabetes type:14, Atrial fibrillation:4  Coronary heart disease:17 |
| Temesgen et al (2018) | Hypertension 38(52.05%), Concurrent infection 23(31.5%)  Congestive heart failure 9(12.32%), Atrial fibrillation 3(4.1%) |
| Zewdie et al (2018) | Hypertension:51 , Diabetes type:11 , Cardiac illness:31 |
